# Unclassifiable Congenital Biliary Dilatation With a Meandering Main Pancreatic Duct and Suspected Pancreaticobiliary Maljunction

**DOI:** 10.7759/cureus.80071

**Published:** 2025-03-05

**Authors:** Yugang Shim, Akinori Sekioka, Shuichi Ota, Tetsuo Ito, Kunihiko Tsuboi

**Affiliations:** 1 Department of Gastrointestinal Surgery, Osaka Saiseikai Noe Hospital, Osaka, JPN; 2 Department of Gastroenterological Surgery, Osaka Saiseikai Noe Hospital, Osaka, JPN

**Keywords:** chronic cholecystitis, congenital biliary dilatation, laparoscopic cholecystectomy (lc), meandering main pancreatic duct, pancreaticobiliary maljunction

## Abstract

Congenital biliary dilatation (CBD) is a congenital disorder in which the bile ducts are locally or diffusely dilated and is specifically defined as a disorder associated with pancreaticobiliary maljunction (PBM). PBM is a congenital anomaly in which the pancreatic and bile ducts merge anatomically outside the duodenal wall. Meandering main pancreatic duct (MMPD) is an anomaly in the shape of the main pancreatic duct (ventral pancreatic duct) in the pancreatic head region that forms an inverted Z-shape or loop shape.

Herein, we present the case of a 30-year-old man who experienced intermittent right upper abdominal pain with an unclassifiable type of CBD, MMPD, suspected PBM, and chronic cholecystitis. Although the patient presented uncommon conditions, we analyzed the pathophysiology and speculated the cause of the symptoms. After laparoscopic cholecystectomy, the patient’s symptoms were relieved. This is the first case report of the rare coexistence of an unclassifiable CBD, MMPD, and suspected PBM.

## Introduction

Congenital biliary dilatation (CBD) is an uncommon congenital dilation of the biliary tree that affects its extrahepatic segments or both intrahepatic and extrahepatic segments at single or multiple sites. The Todani classification is widely used to classify CBD [1,2]. However, there have been several reports of an uncommon type of biliary dilation that does not fit any of the Todani classification types [3].

CBD is often associated with pancreaticobiliary maljunction (PBM), with both conditions affecting each other and causing various symptoms [4]. Although clinical practice guidelines for PBM have provided some recommendations for the treatment strategy, the management of an unclassified type of CBD with PBM remains debatable [5].

Meandering main pancreatic duct (MMPD) is an anomaly in the shape of the main pancreatic duct (ventral pancreatic duct) in the pancreatic head region, forming an inverted Z-shape or loop shape [6]. This is a relatively new concept, and the relationship between CBD and MMPD remains unclear. In this report, we present the case of a patient with CBD, which was difficult to categorize according to the Todani classification, suspected PBM, and MMPD.

## Case presentation

**Figure 1:**
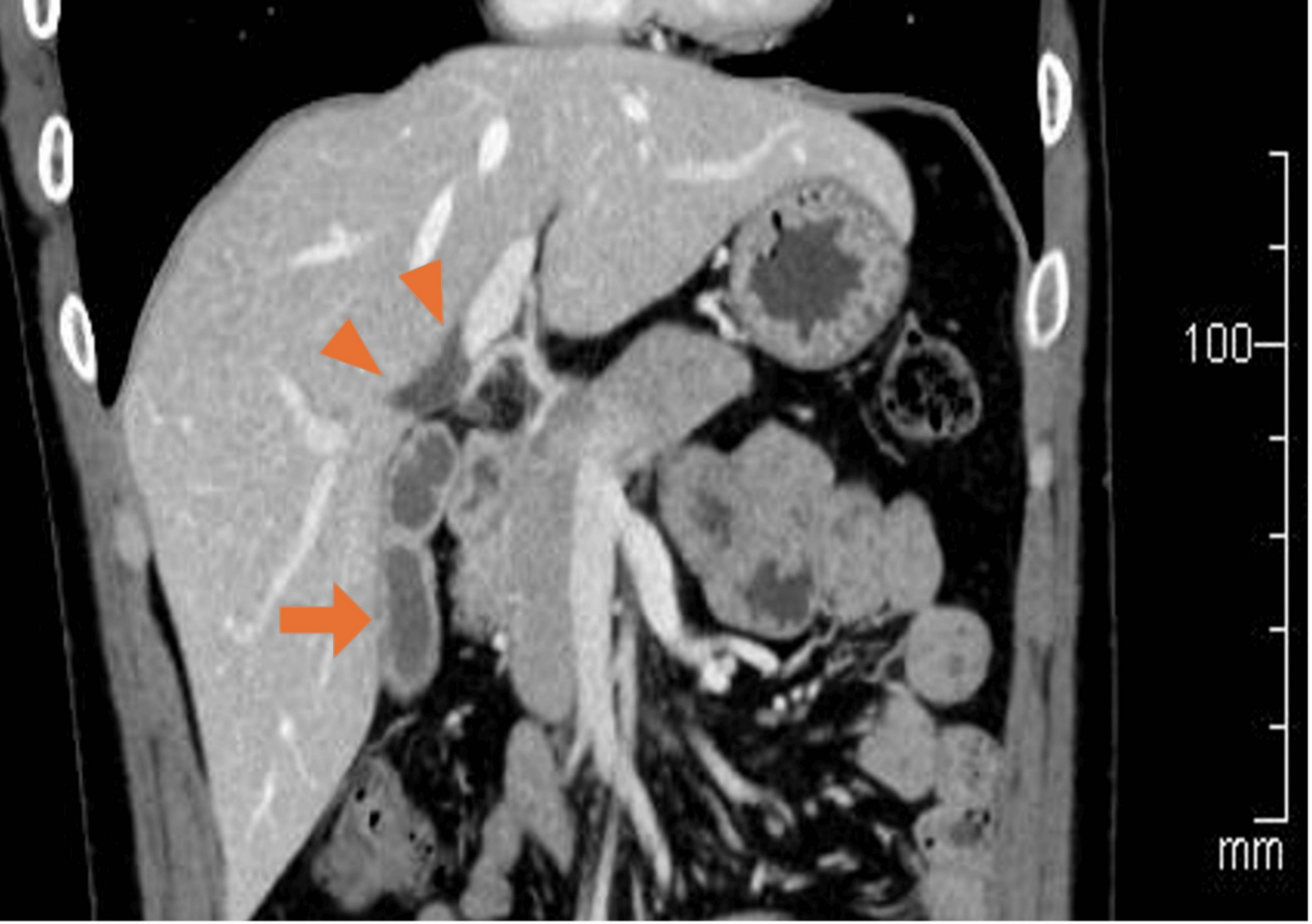
Computed tomography image showing a thickening of the gallbladder wall (arrow) and the dilated bile ducts in the hilar area (arrowheads)

**Figure 2:**
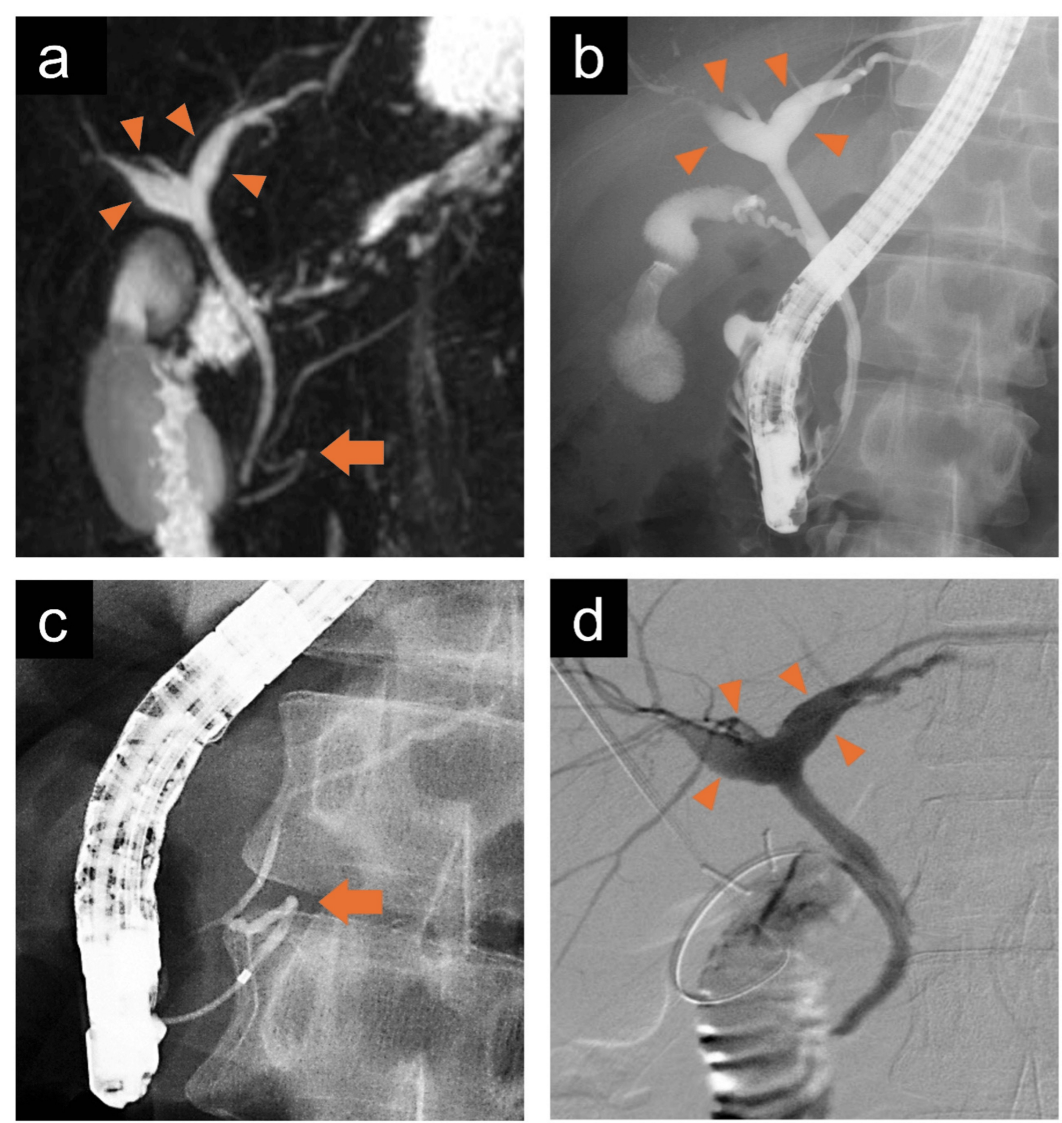
(a) Magnetic resonance cholangiopancreatography image showing the dilation of the right and left hepatic ducts in the hilar area (arrowheads). The fusion site between the ventral and dorsal pancreatic ducts appears flexed (arrow). (b) Endoscopic retrograde cholangiopancreatography showing mildly dilated hepatic ducts in the hilar area (arrowheads) and (c) an abnormally curved main pancreatic duct in the pancreatic head region (arrow). (d) Intraoperative cholangiography showing a dilation of the hilar bile duct (arrowheads). No obvious finding of pancreaticobiliary maljunction is noted

A 30-year-old man presented to our department with intermittent right upper abdominal pain occurring every few months for 10 years. He had a history of taking sodium valproate for epilepsy. He smoked approximately 20 cigarettes per day and had no history of alcohol consumption. Laboratory tests revealed neither inflammatory reactions nor elevated hepatobiliary enzyme levels. Contrast-enhanced computed tomography (CECT) showed a thickening of the gallbladder wall, suggesting chronic cholecystitis. Additionally, the right and left hepatic ducts in the hilar area were dilated, with a maximum diameter of 8 mm; however, the dilation of the extrahepatic bile duct was not significant (Figure 1). Similar to the CECT findings, magnetic resonance cholangiopancreatography (MRCP) showed dilation of the hilar bile duct. Furthermore, MRCP revealed flexed fusion between the ventral and dorsal pancreatic ducts (Figure 2a). Endoscopic retrograde cholangiopancreatography (ERCP) revealed the same findings, with mildly dilated hepatic ducts in the hilar area and an abnormally curved main pancreatic duct in the pancreatic head region (Figures 2b, 2c). During the ERCP, the collected bile showed an amylase level of 6,311 international units (IU)/L, exceeding the normal value of less than 1,000 IU/L [7]. Meanwhile, the lipase level measured 16,000 IU/L, for which the normal range remains undefined.

Based on these findings, diagnoses of CBD (an unclassifiable type according to the Todani classification), suspected PBM, and MMPD (inverted Z-shape) were made. Since the cause of the abdominal pain was presumed to be chronic cholecystitis due to the reflux of pancreatic juice into the gallbladder, we performed laparoscopic cholecystectomy and intraoperative cholangiography. The amylase and lipase levels in the gallbladder bile collected during the operation were 6,328 IU/L and 12,080 IU/L, respectively. Intraoperative cholangiography showed dilation of the hilar bile duct; however, there were no obvious findings indicative of PBM (Figure 2d). The macrofindings of the liver were normal. The operative time was 202 minutes. The postoperative course was uneventful, and the patient was discharged on postoperative day 4. Histopathological examination revealed chronic cholecystitis but no malignant disease. In the clinical follow-up at one year, there were no abdominal symptoms.

## Discussion

CBD is a congenital disorder in which the bile ducts are locally or diffusely dilated, and it is specifically defined as a disorder associated with PBM. The Todani classification is generally used for classifying CBD [1], and some recent studies have reported atypical and uncategorized CBD [3].

PBM is also a congenital disease in which the pancreatic and bile ducts merge anatomically outside the duodenal wall. The mutual reflux of pancreatic juice and bile causes various pathological conditions, such as cholecystopathy, cholangitis, pancreatitis, and intrahepatic stones. Additionally, the continuous reflux of pancreatic juice into the biliary tract damages the bile duct epithelium and can cause biliary tract cancer.

According to the diagnostic criteria for CBD, this entity is specifically defined as biliary dilatation in conjunction with PBM. In other words, it refers to “bile duct dilatation with pancreaticobiliary confluence abnormality,” which includes Todani type I-a, type I-c, and type IV-a. The definition of biliary dilatation does not specify the diameter of the intrahepatic bile ducts but that of the common bile duct (≥6.4 mm) [8]. In this case, mild dilation of the hilar hepatic ducts was detected using various modalities, such as CECT, MRCP, and ERCP. The hilar hepatic bile ducts were locally dilated (8 mm); however, they were difficult to categorize according to the Todani classification. Therefore, this type of intrahepatic biliary dilation would be considered an unclassified and rare form of CBD.

In previous reports, unclassifiable types of CBD were categorized as Todani classification type ID, type Ⅵ, or type Ⅶ [3,9,10]. The choledochal cyst of the cystic duct is described as type Ⅵ, while types ID and Ⅶ are considered subtypes of CBD in the hilar area. However, the cyst of type ID and Ⅶ is relatively large and round shaped, which is different from mild and local dilation of both hepatic ducts in the hilar area in this case. Caroli's disease is classified as type Ⅴ and is characterized by the presence of dilatation confined to the intrahepatic bile ducts [11]. Moreover, it is generally characterized by multiple and focal lesions, which is different from the presentation of this case.

The diagnostic criteria for PBM are based on either imaging tests or anatomical examinations that confirm a long common duct and abnormal confluence on direct contrast imaging. Additionally, abnormally high levels of pancreatic enzymes in the bile ducts or gallbladder have been mentioned as the criteria for an auxiliary diagnosis [12,13]. In this case, neither an abnormally long common channel nor an abnormal union between the pancreatic and bile ducts was detected on contrast imaging. However, we detected abnormally high levels of pancreatic enzymes in the bile ducts and gallbladder. Although occult pancreatobiliary reflux or high confluence of pancreaticobiliary ducts can be a differential diagnosis, this elevation of pancreatic enzymes may be suggestive of PBM and correlated with an abnormal appearance of intrahepatic dilation [14].

In terms of the surgical strategy for PBM with biliary dilation, cholecystectomy and resection of the extrahepatic bile duct are recommended. However, cholecystectomy alone is recommended for PBM without biliary dilation [5]. In this case, there was no guideline-recommended treatment because the patient presented a rare form of right and left hepatic duct dilation in the hilar area. Considering the treatment guidelines for CBD and PBM, we concluded that cholecystectomy alone would suffice because it was expected to relieve the symptoms and prevent the risk of gallbladder cancer.

MMPD is an anomaly in the shape of the main pancreatic duct (ventral pancreatic duct) in the pancreatic head region, forming an inverted Z-shape or loop shape, which is considered related to idiopathic recurrent acute pancreatitis. This pancreatic variant was reported in some previous case reports in combination with a choledochal cyst or intraductal papillary mucinous neoplasm [15,16]. Compared to pancreatic divisum (PD), the most common anatomical variant of the pancreas, MMPD is a relatively rare pancreatic duct malformation [17]. Although the relationship between PD and PBM has been suggested, no reports have described the coexistence of PBM and MMPD [18].

The PubMed database was searched for studies published up to December 2024, using the keywords “congenital biliary dilation” or “choledochal cyst” and “meandering pancreatic duct.” Based on our findings, this is the first case report of the coexistence of unclassifiable CBD and MMPD. Because there are only a few reports in the literature regarding the complications of CBD and MMPD, the relationship between these entities remains unclear. A few previous studies suggested long-term follow-up for patients with pancreaticobiliary reflux or PBM after biliary diversion operation by image studies or measuring serum tumor markers because their entities would be considered as a risk factor for bile duct carcinoma [19,20]. Based on those reports, we believe that periodic follow-up is necessary for this rare condition.

## Conclusions

We encountered a rare case presenting unclassifiable CBD, suspected PBM, and MMPD. Based on the CBD and PBM guidelines, laparoscopic cholecystectomy alone was selected as the treatment for chronic cholecystitis. Since the relevance of CBD, PBM, and MMPD remains unclear, further research is warranted.
